# Recurrent Sepsis Exacerbates CD4^+^ T Cell Exhaustion and Decreases Antiviral Immune Responses

**DOI:** 10.3389/fimmu.2021.627435

**Published:** 2021-02-25

**Authors:** Wanxue He, Kun Xiao, Jiaruo Xu, Wei Guan, Sheling Xie, Kaifei Wang, Peng Yan, Min Fang, Lixin Xie

**Affiliations:** ^1^ College of Pulmonary and Critical Care Medicine, Chinese People’s Liberation Army (PLA) General Hospital, Beijing, China; ^2^ Institute of Microbiology, Chinese Academy of Sciences, Beijing, China

**Keywords:** sepsis, adaptive immune system, recurrent sepsis, immune dysfunction, T cell exhaustion, secondary virus infection

## Abstract

Sepsis is a life-threatening organ dysfunction caused by a dysregulated host response to an infection. It is a disease with a high incidence, mortality, and recurrence rate and frequently results in its survivors requiring readmission into hospitals. The readmission is mainly due to recurrent sepsis. Patients with recurrent sepsis are more susceptible to secondary infections partly due to immune dysfunction, leading to a higher mortality in the long term. However, there remains a gap in the understanding of immunological characteristics and underlying mechanisms of recurrent sepsis. In this study, we used mouse models of acute and recurrent sepsis to investigate their different immunological characteristics. And then we subjected the two mouse models to a secondary influenza A virus (H1N1) infection and characterized the different immune responses. Here, we demonstrated that CD4^+^ T cells present an exacerbated exhaustion phenotype in response to recurrent sepsis as illustrated by the decreased frequency of CD4^+^ T cells, reduced co-stimulatory CD28 and increased inhibitory PD-1 and Tim-3 expression on CD4^+^ T cells, increased frequency of regulatory T cells, and reduced MHC-II expression on antigen-presenting cells. Moreover, we showed that antiviral immune responses decrease in the recurrent sepsis mouse model subjected to a secondary infection as illustrated by the reduced pathogen clearance and inflammatory response. This may be a consequence of the exacerbated CD4^+^ T cell exhaustion. In summary, recurrent sepsis exacerbates CD4^+^ T cell exhaustion and decreases antiviral immune responses, contributing to significant morbidity, increased late mortality, and increased health care burden in recurrent sepsis patients.

## Introduction

Sepsis is a life-threatening organ dysfunction caused by a dysregulated host response to an infection defined by the Third International Consensus Definitions for Sepsis and Septic Shock (Sepsis-3) (1). Sepsis is one of the most critical infectious illnesses, and it has also been declared as a global health priority by the World Health Organization (2). The number of severe sepsis cases in the United States of America exceeded 750,000 in 1995 and increased by 71% in 2007 (3, 4). There were an estimated 48.9 million cases of sepsis and 11.0 million sepsis-related deaths in 2017 across 195 countries and territories (5), suggesting that sepsis has had an extraordinary impact throughout the world. Although more than 30 clinical trials have aimed to reduce sepsis-related mortality, sepsis still contributes to almost 20% of deaths annually in the world due to the absence of effective therapies (6).

In addition to the high incidence and mortality rate of sepsis, individuals that have recovered from sepsis usually require health care after hospital discharge, resulting in an increased readmission and higher mortality rate in the long term (7). It has been reported that approximately 23.4% of sepsis survivors experienced an unplanned 30-day readmission compared with the 10.1% of non-sepsis survivors (8), and this fraction rises to almost one third at 28–90 days from recovery (9). Moreover, recurrent sepsis is one of the most common causes of hospital readmission after the first episode of sepsis. Sepsis contributes to approximately half of the readmissions within 90 days of the first admission (9). In an age- and sex-matched cohort of sepsis survivors, the risk of recurrence was more than eight folds higher than that of developing it for the first time (10). In addition, recurrent sepsis accounted for more than 30% of the deaths, which was significantly higher than, both, patients who were never readmitted and patients readmitted for non-sepsis diagnoses (10, 11). Recurrent sepsis or sepsis recidivism leads to significant morbidity, increased late mortality, and increased health care burden. Despite this, there is still a lack of studies investigating relevant mechanisms of recurrent sepsis.

The development of a deeper understanding of sepsis and the progress in resuscitative strategies have helped recognize immune imbalance as a key pathophysiological change in sepsis (12). The host immune response during and after sepsis is complex and crucially important considering a race to the death between pathogens and the host immune system. Although the timeline and dynamics of the host immune response to sepsis remain debated, it is widely accepted that both pro-inflammatory and anti-inflammatory responses occur early and simultaneously in sepsis (13, 14). However, the net initial effect of these competing processes typically manifests in a dominant hyperinflammatory phase. If sepsis persists, the dysfunction of both the innate and adaptive immune systems induces a marked immunosuppressive state, making patients more susceptible to secondary infections and leading to poorer outcomes (15). Although the risk of recurrent sepsis remains poorly understood, the underlying mechanisms for the recurrence of sepsis may include persistent organ dysfunction (16), gut microbiome dysbiosis (17), ongoing subclinical inflammation (18), and immune dysfunction (7, 15, 19). Generally, all parts of the host defense system, including the innate and adaptive immune systems, coordinate to eliminate invasive pathogens and protect the host from infections. Thus, immune dysfunction plays a prominent role in predisposing sepsis survivors to infection, recurrent sepsis, and associated death.

Although all components of the host immune system are affected significantly during sepsis, alterations in adaptive immune cells, especially T cells, are particularly noteworthy. T cells are specifically activated by pathogens or antigen-presenting cells and subsequently initiate potent immune responses to combat infections. In addition, these cells undergo vigorous proliferation and provide increased protection upon reencountering the pathogen. Notably, T cells play a central role in mediating anti-infection immunity by orchestrating effective immune responses and influencing both innate and adaptive immune cells *via* cytokine production and cell-to-cell communications (20). Numerous studies have investigated the effects of sepsis on T cells, assessing changes in number, phenotype, and function. Sepsis induces an increase in apoptosis of T cells, which is closely associated with increased mortality (19), and disrupts the balance between different T cell subgroups (21, 22). Moreover, studies involving sepsis mouse models and patients with sepsis have reported increases in the expression of coinhibitory receptors, such as programmed cell death protein-1 (PD-1), TNF-related apoptosis-inducing ligand (TRAIL), B and T lymphocyte attenuator (BTLA), and lymphocyte activation gene-3 (LAG-3), in T cells (23–25), partly explaining the persistent reduction in proliferative capacity and inflammatory cytokine production. In addition, not only do the number of Tregs increase during sepsis, their suppressive effects are also amplified (26, 27). Consequently, these alterations result in T cells exhibiting an anergic or exhausted profile, which is closely related to an increased risk of secondary infections and a higher mortality rate during sepsis. However, there is a lack of studies that have investigated the alteration of T cells in recurrent sepsis. Understanding the underlying mechanisms of immune dysfunction following recurrent sepsis is critical for the development of immunotherapies and improving the prognosis for patients of recurrent sepsis.

Therefore, the purpose of this study was to investigate the immunological characteristics and the underlying mechanisms of recurrent sepsis. In the present study, we used mouse models of both acute and recurrent sepsis to investigate their different immunological characteristics, and then we subjected the two mouse models to a secondary viral infection to characterize the different immune responses. Our results provide evidence showing that recurrent sepsis exacerbates CD4^+^ T cell exhaustion and decreases antiviral immune responses, contributing to significant morbidity, increased late mortality, and increased health care burden in recurrent sepsis patients.

## Materials and Methods

### Mice

Female BALB/c mice (7–9 weeks of age) were purchased from Vital River, China. All mice were housed in an animal facility under specific pathogen-free conditions. For virus infection experiments, mice were transferred to a Biosafety Level 2 room in Institute of Microbiology, Chinese Academy of Sciences. Experiments and protocols involving animals were approved by the Regulation of the Institute of Microbiology, Chinese Academy of Sciences (IMCAS) of Research Ethics Committee (permit no. SQIMCAS2018046). All mouse experimental procedures were performed in accordance with the Regulations for the Administration of Affairs Concerning Experimental Animals approved by the State Council of People’s Republic of China.

### Induction of Sepsis

Sepsis in mice was induced by intraperitoneally (i.p.) injecting 0.5 mg/ml of lipopolysaccharide (LPS; serotype 055: B5, Sigma #L2880), dissolved in saline, at a dose of 10 mg/kg. Control mice were intraperitoneally injected with saline at the same dose. To evaluate the difference between acute sepsis and recurrent sepsis, we constructed two different sepsis models. Acute sepsis (AS) was induced by a single LPS stimulation, to trigger the acute response to a severe infection, and were euthanized at 12, 24 and 48 h after LPS or saline administration. Recurrent sepsis (RS) was induced by treatment with three LPS stimulations, once every 5 days to trigger clinically relevant recurrent sepsis, and were sacrificed 5 days after the last injection.

### Virus and Infection

To explore the differences in the anti-infection immune responses between acute and recurrent sepsis mice, we subjected the mice to a secondary virus infection. The mouse-adapted influenza A/Puerto Rico/8/34 (H1N1; PR8) strain was propagated in the chorio-allantoic cavities of 10-day-old specific pathogen-free (SPF) embryonated chicken eggs (Beijing Merial Vital Laboratory Animal Technology) for 48–72 h at 37°C. Allantoic fluids were harvested, aliquoted, and stored at -80°C. Virus titers were determined by plaque assays on Madin–Darby canine kidney (MDCK) cells (ATCC CCL-34). For infection experiments, mice were transferred to a Biosafety Level 2 room. Mice were intraperitoneally (i.p.) anaesthetized with pentobarbital sodium (50 mg/kg body weight) and inoculated intranasally (i.n.) with 1 x 10^3^ pfu PR8 in 40 μl sterile PBS. Control mice were given an equal volume of PBS i.n. for mock infection. Mice were sacrificed at 7 days post infection (dpi).

### Isolation of Splenic Mononuclear Cells

Spleen tissues were collected from the sacrificed mice and processed individually. Single cell suspensions were obtained under sterile conditions by gentle mechanical dissociation of the spleen tissue, in PBS containing 2% fetal bovine serum (FBS), and passing it through a 200-mesh screen. After hemolysis with 1X RBC lysis buffer (Invitrogen, Carlsbad, CA), cells were washed and resuspended in complete RPMI medium, consisting of RPMI 1640 medium supplemented with 10% FBS, 10 mM of 4-(2-hydroxyethyl)-1-piperazineethanesulfonic acid (HEPES), 1× Minimum Essential Medium with non-essential amino acids (MEM-NEAA), 2 mM L-glutamine, 50 mg streptomycin ml^-1^, 50 U penicillin ml^-1^ (all from Gibco, Thermo-fisher Scientific), and 50 mM 2-mercaptoethanol (AMRESCO, Inc.). The number of viable cells was counted by Trypan blue exclusion using a hemocytometer.

### Flow Cytometric Analysis

For flow cytometric analysis of splenic mononuclear cells, up to 2 × 10^6^ cells were added to a 96-well plate. The isolated cell suspension was washed with PBS, incubated with Fcγ receptor blocker CD16/32 (clone 2.4G2; BD Pharmingen), and stained with specific monoclonal antibodies at the indicated concentration in PBS solution containing 1% FBS. The following antibodies were used: fluorescently-conjugated antibodies (BD Pharmingen) against mouse CD3 (145-2C11), CD4 (GK1.5), CD8 (53-6.7), CD19 (1D3), CD25 (3C7), Foxp3 (R16-715), CD69 (H1.2F3), PD-1 (J43), Tim-3 (5D12), and CD28 (37.51). Surface antibody staining was performed at 4°C for 30 min in the dark. For Tregs staining, the cells stained with surface molecules were fixed, permeabilized, and stained for intracellular Foxp3 using the Transcription Factor Buffer kit according to the manufacturer’s instructions (BD Pharmingen). After staining, the cells were washed three times with PBS, and fixed with 0.5% paraformaldehyde at 4°C. The stained cells were analyzed on a FACS Aria III flow cytometer (BD Biosciences). The acquired data was analyzed with FlowJo software (Tree Star).

### Plaque Assay

Virus titers in the lung tissue were determined by plaque assays on Madin-Darby canine kidney (MDCK, ATCC, and CCL-34) cells. Briefly, serial dilutions of the virus were used to infect confluent MDCK cells in 12-well plates for 1 h at 37°C. The virus inoculums were removed by washing with PBS. The cell monolayers were overlaid with an agar medium (DMEM supplemented with 1% low melting point agarose and 1 μg/ml N-tosyl-L-phenylalanyl chloromethyl ketone-treated trypsin) and incubated at 37°C for 48–72 h. The plates were fixed with 4% paraformaldehyde for 1 h, and then the agarose overlays were carefully removed. The wells were incubated with staining buffer (0.1% crystal violet and 20% ethanol in water) for at least 10 min, and was subsequently aspirated. The number of plaques were counted and used to calculate the virus titers.

### Histopathology

Infected mice were sacrificed at 7 dpi. For histological analysis, the lung tissues were removed and fixed with 4% paraformaldehyde for at least 12 h. Post fixing, the tissues were dehydrated in a series of graded alcohols and embedded in paraffin. The tissue was sectioned into 5 μm thick sections, mounted on glass slides, and stained with hematoxylin and eosin solution (H&E). Histological sections were examined using a Zeiss Axio Imager M1 microscope equipped with an AxioCam HRc camera under control of AxioVision 4 software. The histological analysis was processed with Image J software.

### Immunohistochemistry

Infected mice were sacrificed at 7 dpi. Lung specimens from mice exposed to various treatments were fixed in 4% paraformaldehyde, dehydrated and embedded with paraffin. The immunohistochemical staining step was briefly described as follows. Four-micron paraffin tissue sections was placed in a 52°C oven for 2 h to melt the paraffin. The paraffin section was dewaxed in a solvent to rehydrate. Rehydrated paraffin sections were subjected to high-temperature antigen retrieval for 90 s in 10 mM sodium citrate buffer, pH 6.0. Endogenous peroxidase activity was blocked by treating with 3% hydrogen peroxide for 10 min. The blocking of nonspecific binding was performed with 10% normal goat serum for 30 min at room temperature. Then followed by the staining with primary antibodies [the following primary antibodies were used: CD4 (rabbit monoclonal, ab16667, 1:200, Abcam), CD8 (rabbit monoclonal, ab217344, 1:300, Abcam)] overnight at 4°C, and then incubated in secondary antibody [enzyme-labeled goat anti-rabbit IgG polymer (PV-6002, ZSGB-BIO)] for 2 h. The tissue was then reacted with 3,3’-diaminobenzidine (DAB, Sigma) to visualize antibody location. The tissue was redyed by hematoxylin, then tissue was dehydrated and sealed. Images of lung slides were obtained on a Nano Zoomer Slide Scanner.

### Cytokine Analysis

For systemic cytokine detection, peripheral blood was collected from the retro-orbital venous plexus. Sera of mice were collected at 7 dpi. Cytokines were measured using the LEGEND plex Mouse Inflammation Panel (BioLegend, CA, USA) according to the manufacturer’s instructions and read on a Luminex 100 (Bio-Rad) or a FACS LSRFortessa flow cytometer (BD Biosciences).

### Statistical Analysis

Statistical analysis was performed using Prism software (GraphPad). All statistical analyses were performed using an unpaired two-tailed Student’s t-test or two-way ANOVA test as applicable. When applicable, results are displayed as mean ± SD. *p* values <0.05 were considered statistically significant.

## Results

### CD4^+^ T Cells and CD4^+^/CD8^+^ T Cell Ratio Decrease in the Acute and the Recurrent Sepsis Mouse Models, Which Are More Prominent in Recurrent Septic Mice

Sepsis can be experimentally induced by i.p. injecting LPS or endotoxins, which is a constituent of the bacterial cell wall. We i.p. injected LPS to induce a mild sepsis state, which is known to enhance survival rate and permit the long-term study of immune system responses in septic mice (28, 29). Besides, this method is highly reproducible regardless of the skill of the researcher or the individuality of the mice. In addition, mice that experience a mild sepsis demonstrate similar symptomatology, including weight loss, piloerection and lethargy, to that of more severe models such as cecal ligation and puncture (CLP) model.

**Figure 1:**
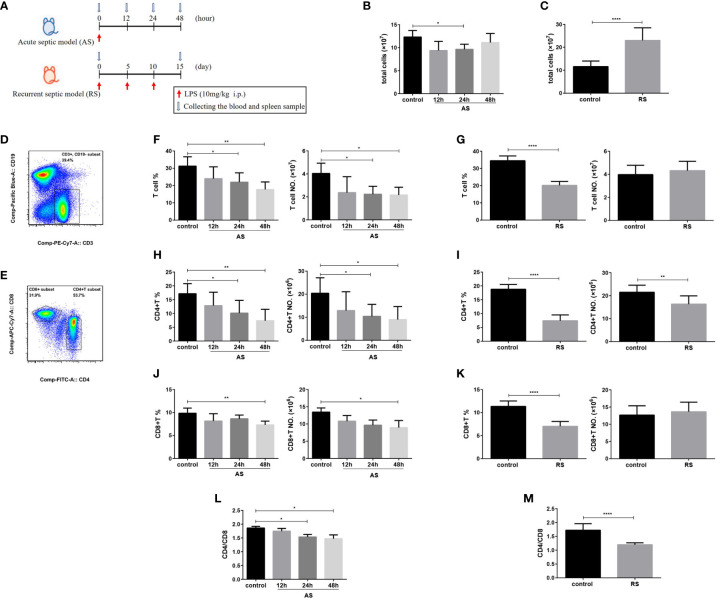
CD4^+^ T cell and CD4^+^/CD8^+^ T cell ratio decrease in the acute and the recurrent sepsis mouse models, which are more prominent in recurrent septic mice. **(A)** Schematic figure of the induction of acute sepsis (AS) and recurrent sepsis (RS) in mice using different frequencies of lipopolysaccharide (LPS) injection. The number of total splenocytes in the AS **(B)** and RS mice **(C)**. Flow cytometry analysis of CD3^+^ CD19^-^ T cells (gated on single cells) **(D)** and CD4^+^ T and CD8^+^ T cells (gated on CD3^+^ CD19^-^ T cells cells) **(E)**. The percentage (left) and number (right) of total T cells **(F)**, CD4^+^ T cells **(H)**, and CD8^+^ T cells **(J)** in the AS model at the indicated time points. The percentage (left) and the number (right) of total T cells **(G)**, CD4^+^ T cells **(I)**, and CD8^+^ T cells **(K)** in the RS model. The CD4^+^/CD8^+^ T cell ratio in the AS **(L)** and RS model **(M)** at the indicated time points. Data are from at least three independent experiments with more than three mice per group in each experiment. Data points indicate means ± SD. *p < 0.05, **p < 0.01, ****p < 0.001.

To investigate the differences of immune dysfunction induced by AS and RS, two mouse models were established using different frequencies of LPS injection (**Figure 1A**). In AS mice, the total splenocyte count showed a significant reduction at 24 h, which recovered by 48 h (**Figure 1B**). However, the total number of splenocytes increased significantly in RS mice (**Figure 1C**). To determine the impact of AS and RS on T lymphocytes, we compared the percentage and absolute numbers of T cells in the spleen at the indicated time points. To this end, we subjected single-cell suspensions off the spleen to flow cytometry. We estimated the percentage and number of T lymphocytes (CD3^+^ CD19^-^), CD4^+^ T lymphocytes (CD3^+^ CD19^-^ CD4^+^), CD8^+^ T lymphocytes (CD3^+^ CD19^-^ CD8^+^), and the CD4^+^/CD8^+^ T lymphocyte ratio in two sepsis mouse models using flow cytometry (**Figures 1D, E** **)**. In AS mice, we found that the percentage and number of total T cells decreased gradually, although the decrease was significant only at 24 and 48 h (**Figure 1F**), and is consistent with previous reports of sepsis-induced lymphopenia (30). In RS mice, although the percentage of total T cells decreased significantly, the absolute number of T cells did not change significantly due to the increase in total splenocyte number (**Figure 1G**). The difference in the percentage and number of CD4^+^ T cells were similar to that of total T cells in AS mice (**Figure 1H**). The percentage and number of CD4^+^ T cells decreased significantly in RS mice (**Figure 1I**). In AS mice, we observed that the percentage and number of CD8^+^ T cells decreased gradually, although the decrease was significant only at 48 h (**Figure 1J**). However, although the percentage of CD8^+^ T cells decreased significantly, the number of CD8^+^ T cells showed no significant difference in RS mice (**Figure 1K**). To further assess immune function, we compared the CD4^+^/CD8^+^ T cell ratio in these two models. We observed that the CD4^+^/CD8^+^ ratio decreased by 20.8% and 30.5% in AS and RS, respectively. A larger difference in the CD4^+^/CD8^+^ ratio, almost reaching one, was seen in RS (**Figures 1L, M** **)**. Together, these results suggest that the percentage and number of CD4^+^ T cells and the CD4^+^/CD8^+^ ratio decreased significantly in AS and RS. It is worth noting that these changes were more prominent in the RS mice, indicating that the immune imbalance was more severe in this model.

### Expression of Cell Surface Receptors, CD69 and CD28, on Splenic CD4^+^ T and CD8^+^ T cells in the Acute and Recurrent Sepsis Mouse Models

**Figure 2:**
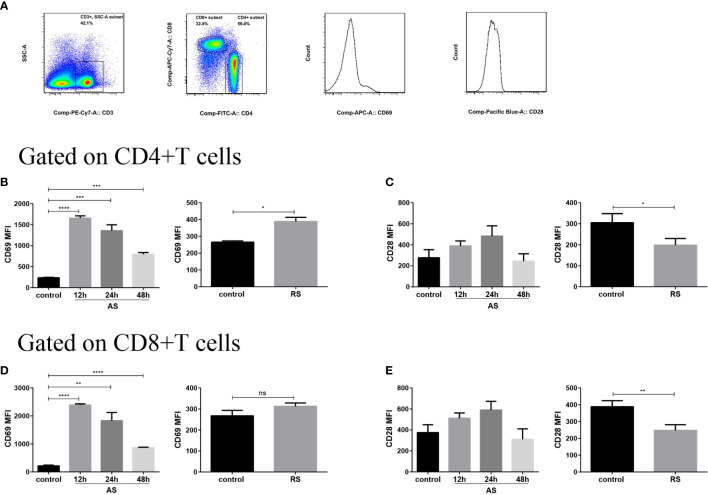
Expression of cell surface receptors, CD69 and CD28, on splenic CD4^+^ T and CD8^+^ T cells in the two sepsis mouse models. **(A)** Flow cytometry analysis of the mean fluorescence intensity (MFI) of CD69 and CD28 expression on gated CD3^+^ CD4^+^ T and CD3^+^ CD8^+^ T cells (gated on single cells) in the two sepsis models. Mean fluorescence intensity (MFI) of CD69 **(B)** and CD28 **(C)** on CD4^+^ T cell subsets in the acute sepsis (AS) (left) and recurrent sepsis (RS) (right) mouse models. MFI of CD69 **(D)** and CD28 **(E)** on CD8^+^ T cell subsets in the AS (left) and RS (right) models. Data are from at least three independent experiments with more than three mice per group in each experiment. Data points indicate means ± SD. *p < 0.05, **p < 0.01, ***p < 0.001, ****p < 0.0001.

To evaluate the receptor expression profiles on splenic T cells, we estimated the expression of cell surface receptors, important to cellular activation, using flow cytometry. We compared the mean fluorescence intensity (MFI) of CD69 and CD28 expression on gated CD4^+^ T and CD8^+^ T cells in the two sepsis models (**Figure 2A**). The expression of early activation marker CD69 increased significantly at 12 h and then declined slowly on both CD4^+^ and CD8^+^ T cell subsets in AS mice, suggesting that both CD4^+^ and CD8^+^ T cell subsets were activated in the early stages of sepsis. However, a moderately weaker accumulation of surface CD69 was observed in CD4^+^ T cells in RS mice, indicating that T cell activation was indeed compromised during RS (**Figures 2B, D** **)**. CD28, a costimulatory molecule that interacts with the CD80/CD86 molecules, is expressed on antigen presenting cells and is critically required for effective T cell activation (31). Although there was no significant difference in the CD28 expression on CD4^+^ T and CD8^+^ T subsets in AS, a significant decrease on both T cell subsets was noted in RS (**Figures 2C, E** **)**. These alterations indicated that both CD4^+^ and CD8^+^ T cell subsets were activated in the early stages of sepsis. Despite this activation in the early stages, RS induced the down regulation of positive costimulatory receptors.

### CD4^+^ T Cells Present Exhausted Phenotype in Recurrent Sepsis

**Figure 3:**
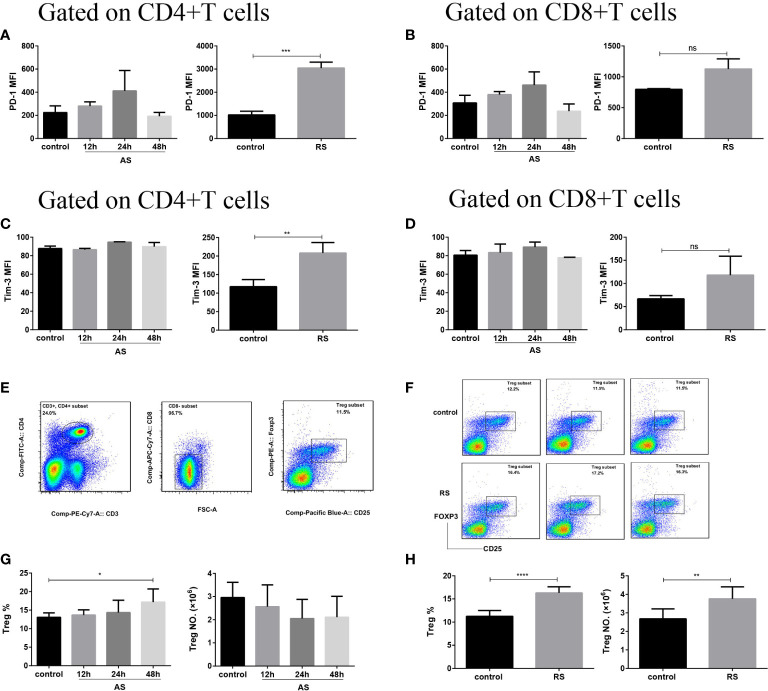
CD4^+^ T cells presented an exhausted phenotype in recurrent sepsis. Mean fluorescence intensity (MFI) of PD-1 on CD4^+^ T cells **(A)** and CD8^+^ T cells **(B)** in the acute sepsis (AS) (left) and recurrent sepsis (RS) (right) mouse models. MFI of Tim-3 on CD4^+^ T cells **(C)** and CD8^+^ T cells **(D)** in the AS (left) and RS (right) mouse models. **(E)** Gating strategy used for the identification of CD3^+^ CD4^+^ CD8^-^ CD25^+^ FOXP3^+^ Tregs. **(F)** Flow cytometric analysis of Tregs in the RS mice (down) and the control mice (up). The percentage (left) and the number (right) of Tregs in AS **(G)** and RS **(H)** model at the indicated time points. Data are from at least three independent experiments with more than three mice per group in each experiment. Data points indicate means ± SD. *p < 0.05, **p < 0.01, ***p < 0.001, ****p < 0.0001.

The accumulation of Tregs and upregulation of inhibitory receptors on T cells are well-known hallmarks of T cell exhaustion (32). To investigate whether T cells undergo sepsis-induced exhaustion in both AS and RS, we estimated PD-1 and Tim-3 expression, on CD4^+^ and CD8^+^ T cell subsets, and the number of Tregs (**Figure 3**). Flow cytometric analysis revealed that both PD-1 and Tim-3 expression significantly increased on CD4^+^ T cells in RS mice, while there was no significant difference on CD8^+^ T cells. In AS mice, both CD4^+^ and CD8^+^ T cells showed no significant difference in PD-1 and Tim-3 expression (**Figures 3A–D**). In addition, both the percentage and absolute number of Tregs (CD3^+^ CD4^+^ CD8^-^ CD25^+^ FOXP3^+^) increased significantly in RS mice. Although the percentage of Tregs increased at 48 h in AS mice, the absolute number of Tregs did not change significantly (**Figures 3E–H**). These results suggested that CD4^+^ T cells presented exhausted phenotype in RS.

### Expression of MHC-II in the Acute and Recurrent Sepsis Mouse Models

**Figure 4:**
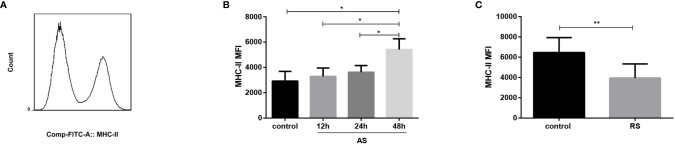
Expression of MHC-II in the acute and recurrent sepsis mouse models. Mean fluorescence intensity (MFI) of MHC-II **(A)** (gated on single cells) in the AS **(B)** and RS **(C)** mouse models. Data are from at least three independent experiments with more than three mice per group in each experiment. Data points indicate means ± SD. *p < 0.05, **p < 0.01.

Although the frequency of both dendritic cells and macrophages was not significantly affected in AS and RS mice (data not shown), we estimated MHC-II expression to evaluate antigen presentation in the two sepsis models. MHC-II molecules present antigens derived from pathogens mainly to CD4^+^ T cells, thereby initiating host defenses, and coordinating innate and adaptive immune responses (33). Mean fluorescence intensity (MFI) of MHC-II expressed on dendritic cells, monocytes, macrophages, and B cells, on gated single cells is shown in **Figure 4A**. We observed that the MHC-II expression increased gradually in AS mice (**Figure 4B**). However, the MHC-II expression decreased significantly in RS mice (**Figure 4C**), suggesting the impaired ability of antigen presenting cells to present antigens to CD4^+^ T cells, exacerbating CD4^+^ T cell exhaustion.

### Recurrent Sepsis Decreases Antiviral Immune Responses

**Figure 5:**
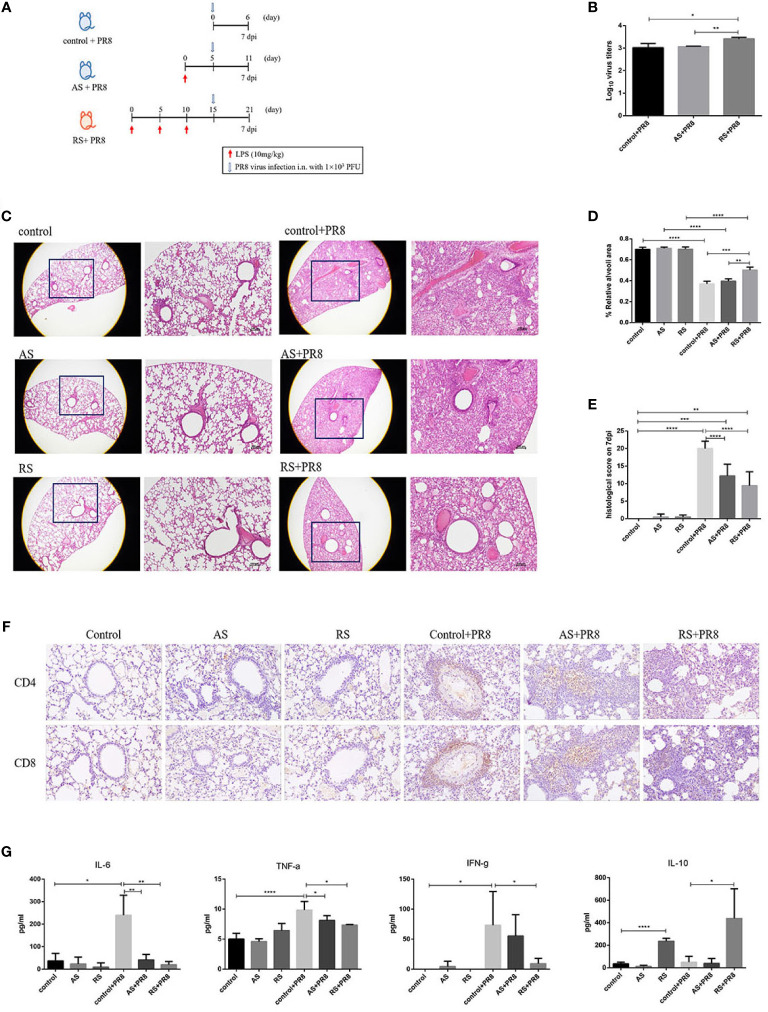
Recurrent sepsis decreased antiviral immune responses. **(A)** Experimental design. Mice received acute sepsis (AS) and recurrent sepsis (RS) stimulation followed by i.n. infection with 10^3^ pfu of PR8 virus. **(B)** Viral titers of infected mice were monitored. **(C)** Hematoxylin-eosin staining (H&E) of lung tissue sections in the control, AS, RS, PR8-infected control (control+PR8), PR8-infected AS (AS+PR8), and PR8-infected RS (RS+PR8) mice (original magnification×10, as labeled; scale bars, 100um). Statistical analysis of relative alveoli area **(D)** and histological scores **(E)** in lung tissue sections from control, AS, RS, control+PR8, AS+PR8, and RS+PR8 mice. **(F)** Representative pictures of CD4 (up) and CD8 (down) immunohistochemical staining in lung tissue sections from control, AS, RS, control+PR8, AS+PR8, and RS+PR8 mice. **(G)** Typical inflammation and anti-inflammation cytokine levels in control, AS, RS, control+PR8, AS+PR8, and RS+PR8 mice. Results are represented as means ± SD. *p < 0.05, **p < 0.01, ***p < 0.001, ****p < 0.0001.

To determine if RS-induced CD4^+^ T cell-exhaustion affects the anti-infective immune function, AS or RS mice were infected with the HIN1 PR8 strain (1x10^3^ PFU/mouse; i.n.) 5 days after the last LPS injection (**Figure 5A**). A significant increase in viral titers was detected in the lungs of PR8-infected RS mice (RS+PR8) compared with that in PR8-infected AS mice (AS+PR8) or in PR8-infected control mice (control+PR8) (**Figure 5B**).

We further performed histological analysis to directly assess lung pathology. As shown in **Figure 5C**, all PR8 infected mice showed prominent leukocyte infiltration at 7 dpi, with a more severe phenotype exhibited by control+PR8 mice and AS+PR8 mice. In the areas with the most severe phenotype, the lung tissue was full of leukocytes, and almost no normal alveolar structures were found in the lungs of these mice. However, RS+PR8 mice showed reduced leukocyte infiltration, pulmonary inflammation, and pathology. The relative alveoli area in the lungs of RS+PR8 mice was significantly higher than that of control+PR8 mice and AS+PR8 mice (**Figure 5D**). Further, we found that the RS+PR8 mice showed an alleviation of pathological features compared with that of control+PR8 mice and AS+PR8 mice (**Figure 5E**).

In addition, we compared the expression of CD4 and CD8 of lung tissue sections using immunohistochemical staining. As shown in **Figure 5F**, the number of CD4^+^ T and CD8^+^ T cells was significantly increased in PR8-infected groups in comparison to non-infected groups. Moreover, the number of CD4^+^ T and CD8^+^ T cells was decreased significantly in RS+PR8 mice in comparison to control+PR8 and AS+PR8 mice, while the decrease of CD4^+^ T cells are more prominent compared to CD8^+^ T cells in RS+PR8 mice.

Additionally, we further estimated the levels of typical inflammation and anti-inflammation cytokine at 7 dpi. As shown in **Figure 5G**, IL-6, TNF-α, and IFN-γ levels were significantly increased in control+PR8 mice compared to that in uninfected mice. Remarkably, the proinflammatory cytokine such as IL-6, TNF-α, and IFN-γ levels decreased significantly in the RS+PR8 mice compared with that in control+PR8 mice and AS+PR8 mice. However, the typical anti-inflammatory cytokine IL-10 increased significantly in RS and RS+PR8 mice. These cytokine level alterations suggested that the proinflammatory responses decreased and the anti-inflammatory responses increased in RS, and these findings are consistent with the exhausted phenotype presented by the CD4^+^ T cells in RS.

In general, we observed that the antiviral immune responses decreased significantly in RS, including increased viral titers, reduced leukocyte infiltration, alleviated pulmonary inflammation and pathology, reduced CD4^+^ T cell infiltration, reduced inflammatory cytokines and increased anti-inflammatory cytokines.

## Discussion

We know that sepsis survivors exhibit an increased readmission and mortality rate commonly due to the development of recurrent sepsis (RS) (9–11). In addition, patients of RS are more susceptible to secondary infections, partly due to immune dysfunction (7, 15, 19). Using experimental “two-hit” mouse models, septic mice have been reported to be at a higher risk of acquiring secondary opportunistic and non-opportunistic infections, such as *Aspergillus*, *Pseudomonas aeruginosa*, *Listeria*, *Candida* and lymphocytic choriomeningitis virus, as a result of a post-sepsis immunosuppressive state (25, 34–36). Despite these observations, there remains a gap in understanding the immune response to secondary infections and the underlying mechanisms of RS. In this study, we used AS and RS mouse models to investigate their different immune characteristics. And then we subjected the two mouse models to a secondary influenza A virus (H1N1) infection and characterized the different immune responses. We are the first to report that recurrent sepsis exacerbates CD4^+^ T cell exhaustion and decreases antiviral immune responses.

Sepsis is induced in mice by the i.p. administration of LPS (28, 29, 37). Although the cecal ligation and puncture (CLP) mouse model of sepsis is regarded as the “gold standard’’ in experimental sepsis research because it closely mimics the clinical course of sepsis, variations in sepsis induction, including the cecal content and skill of researchers, make data comparisons and drawing inferences and conclusions challenging (38, 39). Unlike the CLP model, the LPS model involves the induction of sepsis by injecting lipopolysaccharides, a constituent of the gram-negative bacterial cell wall, and effectively mimics clinically relevant recurrent sepsis using a reproducible and minimally invasive method (40, 41).

We observed a significant decrease in the number of splenocytes at 24 h in AS (**Figure 1B**), corroborating previous reports found by Sharma et al. (42) and Yoon et al. (43). This suggests that both CLP and LPS induces immune cell depletion, which is an important characteristic of sepsis-induced immunosuppression. However, the total number of splenocytes increased significantly in RS mice (**Figure 1C**), probably due to the recurrent attacks of inflammation.

Although almost all components of the host immune system are affected significantly by sepsis, the changes associated with T cells are particularly noteworthy, since they play a central role mediating anti-infective immunity (20). Our findings showed that both the percentage and number of T cells decreased gradually in AS (**Figure 1F**), which is consistent with previous reports of sepsis-induced lymphopenia due to increased apoptosis observed by Unsinger J et al. and Yoon et al. (30, 43). Although the percentage of T cells decreased, the numbers of T cells showed no significant difference between RS and control mice due to an increase in the total splenocyte number in RS mice (**Figure 1G**). Further, we showed an imbalance in the subsets of T cells. As observed in our study, similar to total T cells, both the percentage and number of CD4^+^ T cells decreased significantly in AS (**Figure 1H**), which is widely accepted. It is worth noting that both the percentage and number of CD4^+^ T cells decreased significantly in RS, in spite of massive increase in splenocyte numbers (**Figure 1I**), suggesting that the reduction of CD4^+^ T cells is remarkably prominent in RS; an effect not seen in CD8^+^ T cells (**Figures 1J, K** **)**. The CD4^+^/CD8^+^ ratio of T cells, is considered to be a good indicator of immune function (44). During sepsis, the reduction of CD4^+^/CD8^+^ ratio is closely related to sepsis-induced immunosuppression (45). Xia et al. reported that the CD4^+^/CD8^+^ ratio in patients of sepsis were significantly lower than those in patients with a non-sepsis diagnosis (46). In addition, Jeong et al. reported that the CD4^+^/CD8^+^ ratio was significantly lower in non-survivors than in survivors of sepsis (47). Furthermore, an increased CD4^+^/CD8^+^ ratio is an indicator of enhanced immunity (48). In our study, the reduction of CD4^+^/CD8^+^ ratio and consequently the immune imbalance was more prominent in RS than in AS (**Figures 1L, M** **)**.

In conjunction with alterations in the percentage and absolute number of immune cells, markers expressed on the surface of immune cells which reflect their function are also significantly altered during sepsis (19). Expression of CD69, an early activation marker of immune cells, increased significantly on both CD4^+^ T and CD8^+^ T cells in AS, suggesting that both these T cell subsets are activated in the early stages of sepsis (**Figures 2B, D** **)**, similar to the results of Bommer et al. and Unsinger et al. (19, 30). However, a moderately weaker accumulation of surface CD69 was observed on CD4^+^ T cells in RS, indicating that T cell activation was indeed compromised during RS. RS also induced the down-regulation of positive costimulatory receptor. CD28 which interacts with the CD80/CD86 molecules on antigen presenting cells and is critically required for effective T cell activation (31). In the absence of CD28 signaling, T cells fail to proliferate and produce IFN-γ and IL-2, resulting in a state of anergy or exhaustion and are unresponsive to immune stimulation (49, 50). However, whether the expression of CD28 changes during sepsis is still debated. In our study, we observed that the expression of CD28 decreased significantly on both CD4^+^ T and CD8^+^ T cells in RS but not in AS (**Figures 2C, E** **)**, suggesting that the responsiveness to immune stimulation of CD4^+^ and CD8^+^ T cells declined in RS. Several studies involving sepsis mice and patients have reported increases in the expression of coinhibitory receptors on T cells, such as PD-1 and Tim-3, that generate inhibitory signals and reduce T cell proliferation and function, partly explaining the persistence of an anergic or exhausted profile of T cells (23, 24, 51). Furthermore, anti-PD-1 therapies have been shown to increase pathogen clearance, restore protective immune responses, and improve survival in mouse models of sepsis (52). However, we found no significant difference in PD-1 and Tim-3 expression in both CD4^+^ T and CD8^+^ T cell subsets in AS mice. This is probably due to the use of different sepsis models and an earlier time point for detection. Remarkably in RS, PD-1 and Tim-3 expression increased significantly on CD4^+^ T cells and exhibited trend towards an increase on CD8^+^ T cells (**Figures 3A–D**), suggesting that CD4^+^ T cells display an exhausted phenotype in RS. In addition, the percentage of CD3^+^ CD4^+^ CD8^-^ CD25^+^ FOXP3^+^ regulatory T cells (Tregs) have been reported to increase in studies involving septic mice and patients attribute to a decrease in other effector T cell populations and not because of changes in the absolute numbers of Tregs (53). Similarly, we observed that the percentage of Tregs increased significantly at 48 h in AS mice (**Figure 3G**), suggesting that Tregs are more resistant to sepsis-induced apoptosis. It is noteworthy that both the percentage and absolute number of Tregs increased significantly in RS mice (**Figure 3H**), resulting in an increase in their suppressive effects and contributing to T-cell exhaustion or anergy. Based on the well-known hallmarks of T cell exhaustion (32), our data suggests that CD4^+^ T cells present an exhausted phenotype in recurrent sepsis.

Despite these intrinsic alterations in the adaptive immune system, sepsis has also been shown to impair the function of antigen-presenting cells, such as dendritic cells and monocytes/macrophages and B cells, by reducing the expression of MHC-II, leading to impaired antigen presentation and suppressed T cell responses (19, 54). MHC-II molecules are required for effective antigen presentation to CD4^+^ T cells and coordination of the innate and adaptive immune responses. In addition, as shown by Monneret et al. and Landelle et al., a decrease in MHC-II expression is an independent predictor of secondary infection and higher mortality, after sepsis (55, 56). However, we only observed a trend towards a decrease in MHC-II expression in the early stage of LPS-induced AS. The expression of MHC-II gradually increased in the late stage of LPS-induced AS (**Figure 4B**). One possible explanation for this observation is that the AS model we choose is not as severe as the CLP mouse model or sepsis patients. The LPS-induced AS model mainly induces acute inflammatory responses and stimulates antigen presenting cells to up-regulate MHC-II molecules to combat infections. Notably, the total MHC-II (expressed on dendritic cells, monocytes and macrophages, and B cells) expression decreased significantly in RS mice (**Figure 4C**), suggesting the impaired ability of antigen presenting cells to present antigens to CD4^+^ T cells, exacerbating CD4^+^ T cell exhaustion.

These alterations in the number of T cell subsets, expression of activatory and inhibitory receptors, and expression of MHC-II molecule contribute to the exacerbated exhaustion of CD4^+^ T cells in RS, and this could explain the increased susceptibility to secondary infections and the higher mortality exhibited by patients of recurrent sepsis. However, there remains a gap in understanding the immune response to secondary infections post RS. Hotchkiss et al. observed that reactivation of viruses, such as cytomegalovirus and herpes simplex virus, was common in sepsis induced immunocompromised patients (57, 58). Furthermore, Umadevi et al. showed that a subsequent challenge with human rhinovirus (HRV), after administration of LPS, resulted in viral RNA persistence and a deficient HRV-induced cytokine response in mice (59). Nevertheless, there is a lack of studies investigating the immune response to secondary influenza A virus (H1N1) infections in RS models. Influenza pandemics cause an estimated 140,000–960,000 hospitalizations and 12,000–79,000 deaths per year in the U.S. (https://www.cdc.gov/flu/about/burden/index.html). Among the various subtypes of influenza, a majority of humans are affected by the type A virus (60). Worldwide, influenza A virus infections result in about 3 to 5 million cases of severe illness, and about 250 000 to 500 000 deaths annually (http://www.who.int/mediacentre/factsheets/fs211/en/). Therefore, we subjected AS and RS mice to secondary influenza A virus (H1N1, PR8 strain) infections to explore their immune responses. Our results showed that the viral titers in lung increased significantly in RS, suggesting an impairment in the ability to clear virus in RS. In addition, lung pathology and pro-inflammatory cytokines levels, such as that of IL-6, TNF-α, and IFN-γ, were lower, and the level of anti-inflammatory cytokine IL-10 was higher in RS+PR8 mice, indicating a decrease in the inflammatory responses to clear PR8, and this is consistent with the increased viral titers. Furthermore, CD4^+^ T cell infiltration was decreased significantly in RS+PR8 mice. Therefore, increased viral titers, reduced leukocyte infiltration and alleviated pulmonary inflammation and pathology, reduced CD4^+^ T cell infiltration, reduced inflammatory cytokines, and increased anti-inflammatory cytokines indicate a decreased antiviral immune response, which may be the consequence of exacerbated CD4^+^ T cell exhaustion, in RS.

There are several limitations in our study, investigating the immunological characteristics and underlying mechanisms of RS and AS using mouse models worth mentioning. Although the LPS-induced sepsis mouse model is widely accepted, reproducible, minimally invasive, and enables the mimicking of clinically relevant recurrent sepsis, pathophysiological changes induced by LPS are different from the clinical course of sepsis, and may contribute to differences between our results and clinical situations. Further, instead of analyzing both innate and adaptive immune systems, undoubtedly better reflecting comprehensive immune status, we focused on the adaptive immune system and examined the alterations in response to RS and AS. In addition to increased PD-1 and Tim-3 expression, several studies involving sepsis mice and patients have also reported increases in the expression of other coinhibitory receptors such as TNF-related apoptosis-inducing ligand (TRAIL), B and T lymphocyte attenuator (BTLA), and lymphocyte activation gene-3 (LAG-3) in T cells (24, 61). Thus, we plan to perform additional investigations to understand the contribution of aforementioned receptors to the persistent exhaustion profile. Moreover, it is important to determine if the exacerbated exhaustion phenotype of CD4^+^ T cells in the RS mice is also seen in humans, due to the difference in immunological characteristics, comorbidities, and therapeutic targets between sepsis mice and patients. In addition, future studies investigating the therapeutic implications of CD4^+^ T cell exhaustion reversal in order to enhance immune function, reduce secondary infections, and improve long-term survival in recurrent sepsis patients need to be executed.

## Conclusion

In summary, our results demonstrate CD4^+^ T cells present an exacerbate exhausted phenotype in RS. The potential mechanisms contributing to this phenotype include decreased frequency of CD4^+^ T cells, reduced expression of co-stimulatory receptor CD28, and increased expression of PD-1 and Tim-3 on CD4^+^ T cells, and increased number of Tregs. In addition, RS also causes a significant reduced MHC-II expression on antigen presenting cells, negatively impacting CD4^+^ T cell activation. Therefore, RS, both directly and indirectly disrupts CD4^+^ T cell function, leading to impaired adaptive immune responses, increased susceptibility to secondary infections and poor prognosis. Moreover, on subjecting RS mice to a secondary infection, we found that recurrent sepsis compromises the host’s adaptive immune system, leading to reduced pathogen clearance and inflammatory responses. Together, our data suggests that recurrent sepsis exacerbates CD4^+^ T cell exhaustion and decreases antiviral immune responses, contributing to the significant morbidity, increased late mortality, and increased health care burden. An increased understanding of the underlying immunological alterations accompanying recurrent sepsis will light the way for new therapeutic interventions and better prognosis.

## Data Availability Statement

The original contributions presented in the study are included in the article/supplementary material. Further inquiries can be directed to the corresponding authors.

## Ethics Statement

The animal study was reviewed and approved by the Regulation of the Institute of Microbiology, Chinese Academy of Sciences (IMCAS) of Research Ethics Committee (permit no. SQIMCAS2018046).

## Author Contributions

LX and MF conceived and coordinated the study. WH and KX performed the study and wrote the manuscript. JX, WG, SX, KW, and PY gave advices about concept and revised manuscript. All authors contributed to the article and approved the submitted version.

## Funding

This work was supported by China National Key Research Program (2018ZX09201013), and China PLA Key Research Program (BLB18J008), National Natural Science Foundation of China (91749112).

## Conflict of Interest

The authors declare that the research was conducted in the absence of any commercial or financial relationships that could be construed as a potential conflict of interest.

## References

[1] Singer M Deutschman CS Seymour CW Shankar-Hari M Annane D Bauer M. The Third International Consensus Definitions for Sepsis and Septic Shock (Sepsis-3). JAMA (2016) 315(8):801–10. 10.1001/jama.2016.0287 PMC4968574 26903338

[2] Reinhart K Daniels R Kissoon N Machado FR Schachter RD Finfer S. Recognizing Sepsis as a Global Health Priority - A WHO Resolution. N Engl J Med (2017) 377(5):414–7. 10.1056/NEJMp1707170 28658587

[3] Angus DC Linde-Zwirble WT Lidicker J Clermont G Carcillo J Pinsky MR. Epidemiology of severe sepsis in the United States: analysis of incidence, outcome, and associated costs of care. Crit Care Med (2001) 29(7):1303–10. 10.1097/00003246-200107000-00002 11445675

[4] Lagu T Rothberg MB Shieh MS Pekow PS Steingrub JS Lindenauer PK. Hospitalizations, costs, and outcomes of severe sepsis in the United States 2003 to 2007. Crit Care Med (2012) 40(3):754–61. 10.1097/CCM.0b013e318232db65 21963582

[5] Rudd KE Johnson SC Agesa KM Shackelford KA Tsoi D Kievlan DR. Global, regional, and national sepsis incidence and mortality, 1990-2017: analysis for the Global Burden of Disease Study. Lancet (2020) 395(10219):200–11. 10.1016/S0140-6736(19)32989-7 PMC6970225 31954465

[6] Kempker JA Martin GS. A global accounting of sepsis. Lancet (2020) 395(10219):168–70. 10.1016/S0140-6736(19)33065-X 31954445

[7] Wang T Derhovanessian A De Cruz S Belperio JA Deng JC Hoo GS. Subsequent infections in survivors of sepsis: epidemiology and outcomes. J Intensive Care Med (2014) 29(2):87–95. 10.1177/08850666?12467162 23753224 PMC4393330

[8] Sun A Netzer G Small DS Hanish A Fuchs BD Gaieski DF. Association Between Index Hospitalization and Hospital Readmission in Sepsis Survivors. Crit Care Med (2016) 44(3):478–87. 10.1097/CCM.0000000000001464 26571185

[9] Guirgis FW Brakenridge S Sutchu S Khadpe JD Robinson T Westenbarger R. The long-term burden of severe sepsis and septic shock: Sepsis recidivism and organ dysfunction. J Trauma Acute Care Surg (2016) 81(3):525–32. 10.1097/TA.0000000000001135 27398984

[10] Shen HN Lu CL Yang HH. Risk of Recurrence After Surviving Severe Sepsis: A Matched Cohort Study. Crit Care Med (2016) 44(10):1833–41. 10.1097/CCM.0000000000001824 27120256

[11] DeMerle KM Royer SC Mikkelsen ME Prescott HC. Readmissions for Recurrent Sepsis: New or Relapsed Infection? Crit Care Med (2017) 45(10):1702–8. 10.1097/CCM.0000000000002626 PMC5600690 28742549

[12] Rubio I Osuchowski MF Shankar-Hari M Skirecki T Winkler MS Lachmann G. Current gaps in sepsis immunology: new opportunities for translational research. Lancet Infect Dis (2019) 19(12):e422–e36. 10.1016/S1473-3099(19)30567-5 31630991

[13] Munford RS Pugin J. Normal responses to injury prevent systemic inflammation and can be immunosuppressive. Am J Respir Crit Care Med (2001) 163(2):316–21. 10.1164/ajrccm.163.2.2007102 11179099

[14] Stearns-Kurosawa DJ Osuchowski MF Valentine C Kurosawa S Remick DG. The pathogenesis of sepsis. Annu Rev Pathol (2011) 6:19–48. 10.1146/annurev-pathol-011110-130327 20887193 PMC3684427

[15] Hotchkiss RS Monneret G Payen D. Sepsis-induced immunosuppression: from cellular dysfunctions to immunotherapy. Nat Rev Immunol (2013) 13(12):862–74. 10.1038/nri3552 PMC4077177 24232462

[16] Guirgis FW Khadpe JD Kuntz GM Wears RL Kalynych CJ Jones AE. Persistent organ dysfunction after severe sepsis: a systematic review. J Crit Care (2014) 29(3):320–6. 10.1016/j.jcrc.2013.10.020 24360598

[17] Prescott HC Dickson RP Rogers MA Langa KM Iwashyna TJ. Hospitalization Type and Subsequent Severe Sepsis. Am J Respir Crit Care Med (2015) 192(5):581–8. 10.1164/rccm.201503-0483OC PMC4595694 26016947

[18] Yende S D’Angelo G Kellum JA Weissfeld L Fine J Welch RD. Inflammatory markers at hospital discharge predict subsequent mortality after pneumonia and sepsis. Am J Respir Crit Care Med (2008) 177(11):1242–7. 10.1164/rccm.200712-1777OC PMC2720087 18369199

[19] Boomer JS To K Chang KC Takasu O Osborne DF Walton AH. Immunosuppression in patients who die of sepsis and multiple organ failure. JAMA (2011) 306(23):2594–605. 10.1001/jama.2011.1829 PMC3361243 22187279

[20] Pepper M Jenkins MK. Origins of CD4(+) effector and central memory T cells. Nat Immunol (2011) 12(6):467–71. 10.1038/ni.2038 PMC4212218 21739668

[21] O’Sullivan ST Lederer JA Horgan AF Chin DH Mannick JA Rodrick ML. Major injury leads to predominance of the T helper-2 lymphocyte phenotype and diminished interleukin-12 production associated with decreased resistance to infection. Ann Surg (1995) 222(4):482–90; discussion 90-2. 10.1097/00000658-199522240-00006 7574928 PMC1234878

[22] Pachot A Monneret G Voirin N Leissner P Venet F Bohe J. Longitudinal study of cytokine and immune transcription factor mRNA expression in septic shock. Clin Immunol (2005) 114(1):61–9. 10.1016/j.clim.2004.08.015 15596410

[23] Guignant C Lepape A Huang X Kherouf H Denis L Poitevin F. Programmed death-1 levels correlate with increased mortality, nosocomial infection and immune dysfunctions in septic shock patients. Crit Care (2011) 15(2):R99. 10.1186/cc10112 21418617 PMC3219369

[24] Spec A Shindo Y Burnham CA Wilson S Ablordeppey EA Beiter ER. T cells from patients with Candida sepsis display a suppressive immunophenotype. Crit Care (2016) 20:15. 10.1186/s13054-016-1182-z 26786705 PMC4719210

[25] Condotta SA Khan SH Rai D Griffith TS Badovinac VP. Polymicrobial Sepsis Increases Susceptibility to Chronic Viral Infection and Exacerbates CD8+ T Cell Exhaustion. J Immunol (2015) 195(1):116–25. 10.4049/jimmunol.1402473 PMC4475506 25980007

[26] Monneret G Debard AL Venet F Bohe J Hequet O Bienvenu J. Marked elevation of human circulating CD4+CD25+ regulatory T cells in sepsis-induced immunoparalysis. Crit Care Med (2003) 31(7):2068–71. 10.1097/01.CCM.0000069345.78884.0F 12847405

[27] Scumpia PO Delano MJ Kelly KM O’Malley KA Efron PA McAuliffe PF. Increased natural CD4+CD25+ regulatory T cells and their suppressor activity do not contribute to mortality in murine polymicrobial sepsis. J Immunol (2006) 177(11):7943–9. 10.4049/jimmunol.177.11.7943 17114466

[28] Li CC Munitic I Mittelstadt PR Castro E Ashwell JD. Suppression of Dendritic Cell-Derived IL-12 by Endogenous Glucocorticoids Is Protective in LPS-Induced Sepsis. PloS Biol (2015) 13(10):e1002269. 10.1371/journal.pbio.1002269 26440998 PMC4595142

[29] Napier BA Andres-Terre M Massis LM Hryckowian AJ Higginbottom SK Cumnock K. Western diet regulates immune status and the response to LPS-driven sepsis independent of diet-associated microbiome. Proc Natl Acad Sci U S A (2019) 116(9):3688–94. 10.1073/pnas.1814273116 PMC6397595 30808756

[30] Unsinger J Kazama H McDonough JS Hotchkiss RS Ferguson TA. Differential lymphopenia-induced homeostatic proliferation for CD4+ and CD8+ T cells following septic injury. J Leukoc Biol (2009) 85(3):382–90. 10.1189/jlb.0808491 PMC2653946 19088177

[31] Chen L Flies DB. Molecular mechanisms of T cell co-stimulation and co-inhibition. Nat Rev Immunol (2013) 13(4):227–42. 10.1038/nri3405 PMC3786574 23470321

[32] Wherry EJ Kurachi M. Molecular and cellular insights into T cell exhaustion. Nat Rev Immunol (2015) 15(8):486–99. 10.1038/nri3862 PMC4889009 26205583

[33] Shindo Y Unsinger J Burnham CA Green JM Hotchkiss RS. Interleukin-7 and anti-programmed cell death 1 antibody have differing effects to reverse sepsis-induced immunosuppression. Shock (2015) 43(4):334–43. 10.1097/SHK.0000000000000317 PMC4359667 25565644

[34] Benjamim CF Hogaboam CM Lukacs NW Kunkel SL. Septic mice are susceptible to pulmonary aspergillosis. Am J Pathol (2003) 163(6):2605–17. 10.1016/S0002-9440(10)63615-2 PMC1892404 14633632

[35] Delano MJ Thayer T Gabrilovich S Kelly-Scumpia KM Winfield RD Scumpia PO. Sepsis induces early alterations in innate immunity that impact mortality to secondary infection. J Immunol (2011) 186(1):195–202. 10.4049/jimmunol.1002104 21106855 PMC3771366

[36] Davis CG Chang K Osborne D Walton AH Dunne WM Muenzer JT. Increased susceptibility to Candida infection following cecal ligation and puncture. Biochem Biophys Res Commun (2011) 414(1):37–43. 10.1016/j.bbrc.2011.09.017 21939638 PMC3195872

[37] Honda T He Q Wang F Redington AN. Acute and chronic remote ischemic conditioning attenuate septic cardiomyopathy, improve cardiac output, protect systemic organs, and improve mortality in a lipopolysaccharide-induced sepsis model. Basic Res Cardiol (2019) 114(3):15. 10.1007/s00395-019-0724-3 30838474

[38] Wynn JL Scumpia PO Delano MJ O’Malley KA Ungaro R Abouhamze A. Increased mortality and altered immunity in neonatal sepsis produced by generalized peritonitis. Shock (2007) 28(6):675–83. 10.1097/SHK.0b013e3180556d09 17621256

[39] Gentile LF Nacionales DC Lopez MC Vanzant E Cuenca A Cuenca AG. Protective immunity and defects in the neonatal and elderly immune response to sepsis. J Immunol (2014) 192(7):3156–65. 10.4049/jimmunol.1301726 PMC3967513 24591376

[40] Opal SM. Endotoxins and other sepsis triggers. Contrib Nephrol (2010) 167:14–24. 10.1159/000315915 20519895

[41] Saito M Inoue S Yamashita K Kakeji Y Fukumoto T Kotani J. IL-15 Improves Aging-Induced Persistent T Cell Exhaustion in Mouse Models of Repeated Sepsis. Shock (2020) 53(2):228–35. 10.1097/SHK.0000000000001352 31935201

[42] Sharma A Yang WL Matsuo S Wang P. Differential alterations of tissue T-cell subsets after sepsis. Immunol Lett (2015) 168(1):41–50. 10.1016/j.imlet.2015.09.005 26362089 PMC4636913

[43] Yoon SJ Kim SJ Lee SM. Overexpression of HO-1 Contributes to Sepsis-Induced Immunosuppression by Modulating the Th1/Th2 Balance and Regulatory T-Cell Function. J Infect Dis (2017) 215(10):1608–18. 10.1093/infdis/jix142 28368519

[44] Yang G Hu RY Deng AJ Huang Y Li J. Effects of Electro-Acupuncture at Zusanli, Guanyuan for Sepsis Patients and Its Mechanism through Immune Regulation. Chin J Integr Med (2016) 22(3):219–24. 10.1007/s11655-016-2462-9 26825083

[45] Mansilla-Rosello A Ferron-Orihuela JA Ruiz-Cabello F Garrote-Lara D Delgado-Carrasco S Tamayo-Pozo F. Interleukin-1beta and ibuprofen effects on CD4/CD8 cells after endotoxic challenge. J Surg Res (1996) 65(1):82–6. 10.1006/jsre.1996.0347 8895611

[46] Xia XJ Liu BC Su JS Pei H Chen H Li L. Preoperative CD4 count or CD4/CD8 ratio as a useful indicator for postoperative sepsis in HIV-infected patients undergoing abdominal operations. J Surg Res (2012) 174(1):e25–30. 10.1016/j.jss.2011.10.006 22225978

[47] Jeong SJ Yoon SS Han SH Yong DE Kim CO Kim JM. Evaluation of humoral immune response to nosocomial pathogen and functional status in elderly patients with sepsis. Arch Gerontol Geriatr (2014) 58(1):10–4. 10.1016/j.archger.2013.07.001 23998496

[48] Wang J Zhou J Bai S. Combination of Glutamine and Ulinastatin Treatments Greatly Improves Sepsis Outcomes. J Inflamm Res (2020) 13:109–15. 10.2147/JIR.S234122 PMC7037133 32110086

[49] Fathman CG Lineberry NB. Molecular mechanisms of CD4+ T-cell anergy. Nat Rev Immunol (2007) 7(8):599–609. 10.1038/nri2131 17612584

[50] Aziz M Yang WL Matsuo S Sharma A Zhou M Wang P. Upregulation of GRAIL is associated with impaired CD4 T cell proliferation in sepsis. J Immunol (2014) 192(5):2305–14. 10.4049/jimmunol.1302160 PMC3943916 24477910

[51] Day CL Kaufmann DE Kiepiela P Brown JA Moodley ES Reddy S. PD-1 expression on HIV-specific T cells is associated with T-cell exhaustion and disease progression. Nature (2006) 443(7109):350–4. 10.1038/nature05115 16921384

[52] Chang KC Burnham CA Compton SM Rasche DP Mazuski RJ McDonough JS. Blockade of the negative co-stimulatory molecules PD-1 and CTLA-4 improves survival in primary and secondary fungal sepsis. Crit Care (2013) 17(3):R85. 10.1186/cc12711 23663657 PMC3706819

[53] Venet F Pachot A Debard AL Bohe J Bienvenu J Lepape A. Increased percentage of CD4+CD25+ regulatory T cells during septic shock is due to the decrease of CD4+CD25- lymphocytes. Crit Care Med (2004) 32(11):2329–31. 10.1097/01.ccm.0000145999.42971.4b 15640650

[54] Cazalis MA Friggeri A Cave L Demaret J Barbalat V Cerrato E. Decreased HLA-DR antigen-associated invariant chain (CD74) mRNA expression predicts mortality after septic shock. Crit Care (2013) 17(6):R287. 10.1186/cc13150 24321376 PMC4056003

[55] Monneret G Lepape A Voirin N Bohe J Venet F Debard AL. Persisting low monocyte human leukocyte antigen-DR expression predicts mortality in septic shock. Intensive Care Med (2006) 32(8):1175–83. 10.1007/s00134-006-0204-8 16741700

[56] Landelle C Lepape A Voirin N Tognet E Venet F Bohe J. Low monocyte human leukocyte antigen-DR is independently associated with nosocomial infections after septic shock. Intensive Care Med (2010) 36(11):1859–66. 10.1007/s00134-010-1962-x 20652682

[57] Hotchkiss RS Monneret G Payen D. Immunosuppression in sepsis: a novel understanding of the disorder and a new therapeutic approach. Lancet Infect Dis (2013) 13(3):260–8. 10.1016/S1473-3099(13)70001-X PMC3798159 23427891

[58] Walton AH Muenzer JT Rasche D Boomer JS Sato B Brownstein BH. Reactivation of multiple viruses in patients with sepsis. PloS One (2014) 9(2):e98819. 10.1371/journal.pone.0098819 24919177 PMC4053360

[59] Sajjan U Ganesan S Comstock AT Shim J Wang Q Nagarkar DR. Elastase- and LPS-exposed mice display altered responses to rhinovirus infection. Am J Physiol Lung Cell Mol Physiol (2009) 297(5):L931–44. 10.1152/ajplung.00150.2009 PMC2777490 19748999

[60] Cohen AL Hellferscee O Pretorius M Treurnicht F Walaza S Madhi S. Epidemiology of influenza virus types and subtypes in South Africa, 2009-2012. Emerg Infect Dis (2014) 20(7):1162–9. 10.3201/eid2007.131869 PMC4073865 24960314

[61] Boomer JS Shuherk-Shaffer J Hotchkiss RS Green JM. A prospective analysis of lymphocyte phenotype and function over the course of acute sepsis. Crit Care (2012) 16(3):R112. 10.1186/cc11404 22742734 PMC3580670

